# Linking plant and soil indices for water stress management in black gram

**DOI:** 10.1038/s41598-020-79516-3

**Published:** 2021-01-13

**Authors:** Afshin Khorsand, Vahid Rezaverdinejad, Hossein Asgarzadeh, Abolfazl Majnooni-Heris, Amir Rahimi, Sina Besharat, Ali Ashraf Sadraddini

**Affiliations:** 1 Department of Water Engineering, Urmia University, Urmia, Iran; 2 Department of Water Engineering, Urmia University, 11 Km Sero Road, Post box: 165, 5756151818 Urmia, Iran; 3 Department of Soil Science, Urmia University, Urmia, Iran; 4 Department of Water Engineering, University of Tabriz, Tabriz, Iran; 5 Department of Agronomy, Urmia University, Urmia, Iran

**Keywords:** Drought, Sensors and probes, Hydrology

## Abstract

Measurement of plant and soil indices as well as their combinations are generally used for irrigation scheduling and water stress management of crops and horticulture. Rapid and accurate determination of irrigation time is one of the most important issues of sustainable water management in order to prevent plant water stress. The objectives of this study are to develop baselines and provide irrigation scheduling relationships during different stages of black gram growth, determine the critical limits of plant and soil indices, and also determine the relationships between plant physiology and soil indices. This study was conducted in a randomized complete block design at the four irrigation levels 50 (I_1_), 75 (I_2_), 100 (I_3_ or non-stress treatment) and 125 (I_4_) percent of crop’s water requirement with three replications in Urmia region in Iran in order to irrigation scheduling of black gram using indices such as canopy temperature (T_c_), crop water stress index (CWSI), relative water content (RWC), leaf water potential (LWP), soil water (SW) and penetration resistance (*Q*) of soil under one-row drip irrigation. The plant irrigation scheduling was performed by using the experimental crop water stress index (CWSI) method. The upper and lower baseline equations as well as CWSI were calculated for the three treatments of I_1_, I_2_ and I_3_ during the plant growth period. Using the extracted baselines, the mean CWSI values for the three treatments of I_1_, I_2_ and I_3_ were calculated to be 0.37, 0.23 and 0.15, respectively, during the growth season. Finally, using CWSI, the necessary equations were provided to determine the irrigation schedule for the four growing stages of black gram, i.e. floral induction-flowering, pod formation, seed and pod filling and physiological maturity, as (T_c_ − T_a_)_c_ = 1.9498 − 0.1579(AVPD), (T_c_ − T_a_)_c_ = 4.4395 − 0.1585(AVPD), (T_c_ − T_a_)_c_ = 2.4676 − 0.0578(AVPD) and (T_c_ − T_a_)_c_ = 5.7532 − 0.1462(AVPD), respectively. In this study, soil and crop indices, which were measured simultaneously at maximum stress time, were used as a complementary index to remove CWSI constraints. It should be noted that in Urmia, the critical difference between the canopy temperature and air temperature (T_c_ − T_a_), soil penetration resistance (*Q*), soil water (SW) and relative water content (RWC) for the whole growth period of black gram were − 0.036 °C, 10.43 MPa and 0.14 cm^3^ cm^−3^ and 0.76, respectively. Ideal point error (IPE) was also used to estimate RWC, (T_c_ − T_a_) and LWP as well as to select the best regression model. According to the results, black gram would reduce its RWC less through reducing its transpiration and water management. Therefore, it can be used as a low-water-consuming crop. Furthermore, in light of available facilities, the farmer can use the regression equations between the obtained soil and plant indices and the critical boundaries for the irrigation scheduling of the field.

## Introduction

In areas where crops are irrigated, proper management and scheduling for optimal water use is essential. As the crop water content is affected by the combined factors of climate and soil water (SW), proper irrigation scheduling should be based on the water content of the crop, the response of plant indices to the SW content and evaporative requirements^1^. In recent years, a wide range of new approaches have been proposed for irrigation scheduling, but they have turned out to be controversial. Many of these approaches are based on the crop reaction rather than on the direct measurement of SW^2^. Leaf water potential (LWP), stomatal resistance, photosynthesis intensity, and canopy temperature (T_c_) are basic indices that show the water content of the crops and serve as a tool for irrigation scheduling for many crops^3,4^.

SW measurement, atmospheric variables, crop measurements or a combination of them are used for the irrigation scheduling of different crops^5^. There are various indices for determining the crop water content and irrigation scheduling, each of which has its advantages and disadvantages as discussed by Jones^2^. Some of these indices are soil-based (such as SW and soil water potential) and some are based on the crop (such as LWP, T_c_, stomatal resistance, leaf color, and photosynthesis intensity). Accurate and timely irrigation scheduling to avoid water stress should be considered as one of the most important issues in sustainable water management. The severity of water stress depends on time and duration. Therefore, specific methods should be developedto properly classify the crop water requirements, taking into account economic and environmental benefits^6^. When the crop is exposed to water deficit, stomatal conductance and latent heat exchange are reduced and the cooling effect of evaporation is reduced, resulting in the plant leaves being warmer than when the crop is not stressed. This property can be used to measure the crop water content after measuring the T_c_ of the plant^7,8^.

T_c_ indicates the crop transpiration intensity and water stress. It has high potential for irrigation scheduling^9^. It is a method that was applied after the development of infrared thermometers for irrigation scheduling. In 1981, an experimental and applied index, Crop water stress index (CWSI), was introduced^10^ that can predict the irrigation time^11,12^.

The difference between the canopy temperature and air temperature (T_c_ − T_a_) was used in the peach garden irrigation management by Wang and Gartung^13^ by using the SW and stem water potential. The crop’s response to environmental conditions is a key factor in the irrigation scheduling and improvement. Irrigation scheduling is traditionally performed by soil, water and climate variables. However, the use of crop-based water stress indices has been widely studied to reduce the risk of tree and crop damage due to water stress, because crop-based indices show the cumulative effects of soil, plant and climate conditions^14^. Researchers investigated the effect of wind speed on the upper and lower baseline (T_c_ − T_a_) as well as the LWP and CWSI under different drought conditions for maize, cotton, bean and sorghum^15^. Sharatt et al.^16^ stated that alfalfa LWP was lower in soil with lower water content, thereby reducing evapotranspiration and increasing the leaf temperature. The lower the LWP, the more water the crop requires. Measuring water potential in crop cells and tissues is one of the most important issues in studying water-crop relationships.

Orta et al.^17^ studied the irrigation time taking into account the SW content as well as the difference in the leaf and environmental temperature. They found a direct correlation between the crop water content and the temperature difference between the leaf and the environment. When the plants experience the slightest water stress, the stomata immediately close, transpiration is reduced, and the leaf temperature increases. Mangus et al.^18^ showed a close correlation between the canopy temperature and the crop’s water use properties. Researchers showed that the crop water stress occurs at the highest solar radiation at noon time. T_c_ was measured at the highest canopy temperature between 12 and 17^19,20^. For the instantaneous calculation of crops water stress, relative humidity, air temperature, solar radiation and crop temperature are combined with thermal camera images^18^.

A recent soil index for irrigation scheduling is soil *Q*^21^. Soil *Q* is the most important soil bulk density trait. As the density of the soil increases, the force required for the tip of the penetrometer to penetrate the soil increases. This force can also influence the root penetration^22^. The present study has sought to, answer the following questions: What is the critical soil *Q* for black gram? What is the role of (T_c_ − T_a_) in the critical soil *Q*? It is important to answer these questions for the irrigation scheduling by different methods^23^. Another question is whether or not the CWSI restrictions can be lifted.

Black gram (*Vigna mungo* L.) is a plant of the Fabaceae family. It is cultivated in West Azerbaijan Province (Iran) in second cropping after wheat harvest. It is a source of income for local farmers. The plant has high nutritional value and may be used to compensate for hidden hunger in poor communities. Like many legumes, black gram contains important nutrients, including essential amino acids, which is insturemntal in improving the diet and enhancing human and animal health, especially in the areas where this plant is cultivated. Therefore, the irrigation schedule of this product is very important in water deficit areas with a view to increasing the water use efficiency.

The research hypotheses are as follows: (a) CWSI is affected by different irrigation regimes; (b) relative water content (RWC) of the leaves is the most accurate method of irrigation scheduling; (c) there is a correlation between (T_c_ − T_a_) and soil *Q*; (d) LWP is affected by soil *Q* and other crop indices. The objectives of the present study are: (a) development of upper and lower baselines and presenting irrigation scheduling relations during different stages of black gram growth; (b) delimitation of critical indices of plant physiology and soil; (c) determining the relationships between plant physiology indices and soil indices.

## Materials and methods

### Study area

The present study was carried out on black gram in at the research division of Urmia University in the 2017 crop year. This farm is located at the latitude of 37° 39′ N and the longitude of 44° 58′ E and at an altitude of 1,365 m above the sea level in northwestern Iran^21^. The climate in Urmia city is semi-arid and cold semi-arid, according to the Embereger and De Martonne Methods. Important growth stages of black gram as well as other operations are presented in Table 1. The dimensions of the plots were 3 m × 2 m and the distance between the plots was 2 m. In the next step, seedlings of black gram were planted on rows 50 cm apart and on rows 10 cm apart. Soil samples were also taken to determine soil physical properties (Table 2).

**Table 1:** Agronomic details and dates for field experiment.

Black gram	Experimental year 2017	Description
Planting population (plants/ha)	200,000	–
Planting date	May-14	The seeds were planted in trays
Initial emergence date	May-21	–
70% emergence date	May-27	–
Transplanting date	Jun-07	After complete emergence
Harvest date	Sep-13	–
The first weeding date	May 25-May 26	Several times during vegetative phase
Fertilization date of macro elements (N, P, K)	May-28	Once before transplanting
Fertilization date of urea	June 21-July 2	–
Spraying date of liquid Fertilizer	Jul-05	Dissolving with 7.2 L water
Spraying date of amino acid fertilizer	Jul-19	Dissolving with 7.2 L water
Application date of water stress	Jul-19	Tenth irrigation

**Table 2:** Physical properties of the experimental soil.

Soil depth (cm)	Particle size distribution (%)			Texture class	FC (cm^3^ cm^−3^)	PWP (cm^3^ cm^−3^)	BD (g cm^−3^)
	Clay (%)	Silt (%)	Sand (%)				
0–30	44	50	6	Silt clay loam	0.353	0.241	1.370
30–60	39	33	28	Clay loam	0.360	0.249	1.473

Clay (< 0.002 mm), silt (0.002–0.05 mm), sand (0.05–2 mm) (USDA classification). FC: field capacity; PWP: permanent wilting point; BD: bulk density.

### Experimental design and irrigation method

**Figure 1:**
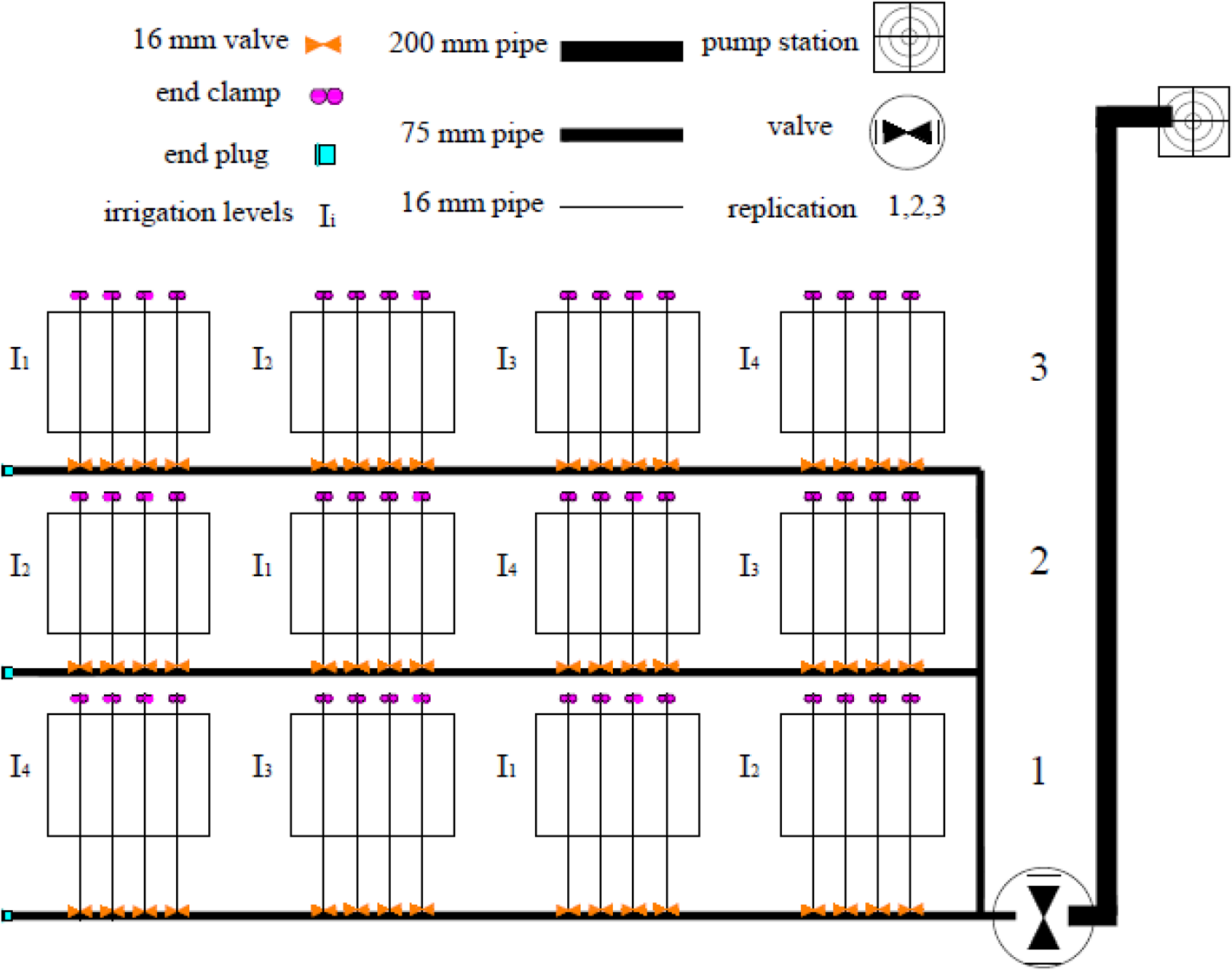
Plot layout of field experiment.

In the present study, the effect of different irrigation water treatments on black grams was investigated. The experimental design was a randomized complete block design with four aqueous treatments in three replications (Fig. 1). Water treatments included 50% (I_1_), 75% (I_2_), 100% (I_3_) or the control treatment, and 125% (I_4_) of crop water requirement. To determine the water requirement, meteorological parameters were obtained from the meteorological station of the Faculty of Agriculture of Urmia University, and the reference evapotranspiration (ET_o_) was calculated by V3.1 ET_o_ Calculator^24^. The equation used in this software to calculate ET_o_ is the FAO-modified Penman–Monteith equation^25^. Using the following equation, the ET_o_ calculated by multiplying the crop coefficient (*K*_*c*_)^26^ was generalized to the potential evapotranspiration (ET_c_) values of black gram (Table 3).

1 $$ET_{c} = K_{c} \times ET_{o}$$

**Table 3:** Black gram irrigation scheduling for control treatment (I_3_), during 2017 growing seasons.

No	Irrigation date	Irrigation depth (mm)	No	Irrigation date	Irrigation depth (mm)
1	11 June	15.62	12	26 July	22.24
2	14 June	11.28	13	30 July	28.60
3	18 June	17.20	14	2 August	21.14
4	21 June	14.84	15	6 August	27.74
5	25 June	18.53	16	9 August	21.27
6	28 June	15.12	17	13 August	28.11
7	2 July	24.57	18	16 August	25.67
8	5 July	18.51	19	20 August	24.57
9	12 July	47.98	20	27 August	47.67
10	19 July	48.36	21	3 September	49.34
11	23 July	30.49	–	–	–

Immediately after transferring the seedling, an irrigation step was carried out to plant the seedlings in the field. The black gram is used as a second crop in the area while water stress was applied in mid-July (tenth irrigation) for optimal plant establishment. The irrigation water amounts for each treatment, ET_c_ and ET_o_, during the crop growth period are presented in Table 4. Plant irrigation was performed during the growing season using a 16 mm dripper pipe located next to each row^21^. The 16 mm dripper pipe had constant pressure and the flow rate of emitters was 4 L h^-1^. At the beginning of each 16 mm pipe, a 16 in 16 mm valve was used to control water stress over time^21^. According to soil analysis, four fertilizer treatments were used to prevent nutrient deficiency^27^ and these four treatments received equal amounts of fertilizers^28^. Fertilizer was used as a spray and soil fertilizers (Table 1).

**Table 4:** Irrigation amounts and ET_o_ (mm), during 2017 growing seasons.

Treatments	Deficit irrigation		Full irrigation	Additional irrigation	ET_c_	ET_o_
	I_1_	I_2_	I_3_	I_4_		
	(50%)	(75%)	(100%)	(125%)		
Total	279.41	419.12	558.82	698.53	502.94	554.70

ET_o_: reference evapotranspiration.

### Weather data

The meteorological parameters of the region including minimum, maximum and mean temperature, minimum and maximum relative humidity, wind speed, precipitation and sunshine hours were obtained during the present study from the Meteorology Station of Urmia University, which was the closest meteorology station to the research area (Table 5). The WatchDog meteorological device installed around the farm was also used to obtain air temperature and relative humidity data over 10 min intervals to calculate air vapor pressure deficit, and the data obtained from this device was inserted by a cable and the SpecWare 9 software into the laptop^21^.

**Table 5:** Average and sum monthly weather parameters, during 2017 growing seasons^21^.

Parameter	Month					
	May	June	July	August	September	October
Air temp._mean_ (^°^C)	18.7	24	27.4	26.9	22.2	14.4
Air temp._max_ (°C)	24.9	31.3	34.5	34.2	29.4	21
Air temp._min_ (°C)	12.5	16.7	20.2	19.6	15.1	7.9
Relative humidity_max_ (%)	65	54	50	52	57	63
Relative humidity_min_ (%)	26	24	25	29	35	35
Wind speed (m s^-1^)	7	5	4	3	4	R
Precipitation (mm)	15.1	0	0	2.2	0	3.3
Hours of bright sunshine (h day^−1^)	309.8	341.9	344.4	349	296.5	255.4

### Plant measurements

#### Infrared thermometers (IRTs)

In this study, the FLUKE Mini IR62 infrared device was used to measure the canopy temperature (T_c_). One of the characteristics of all infrared devices is a special feature called field of view. The field of view is the maximum angle between the rays coming from the object being measured (the leaves of the black gram in the present study) received by the device. The larger the field of view, the larger the image size measured by the device. Furthermore, the greater the distance of the device to the target being measured the greater the field of view. Therefore, the field of view of the device is expressed as D:S which is the ratio of the object diameter (point size or object diameter) to the distance from the device. The D:S ratio of the infrared thermometer in the present study was 10:1.

T_c_ of black gram was not measured from planting to 10^th^ July due to its small size and field of view of the device^12^. T_c_ was measured from four geographical directions for each treatment (with three replications) when it was sunny and cloudless^6,28^. Measurements were made on different leaves of black gram at an angle of 30° to 45° to the horizon and an average T_c_ was obtained from an average 12 readings for each treatment^28^. Generally, for each treatment in a single day, 84 T_c_ readings were obtained in 7 h (8:50 to 14:50). To obtain the lower baseline equation of the method of Idso et al.^10^ (Eq. (2)), T_c_ of black gram was measured for control treatment (I_3_) from 8:50 to 14:50 in the post-irrigation days^6^. Meantime, in a bid to determine the experimental CWSI (Eq. (5)) and the calculation of (T_c_ − T_a_)_m_, T_c_ of black gram was measured from 11:50 to 14:50 in the pre-irrigation days for all three treatments (I_1_, I_2_ and I_3_). CWSI values were calculated for the four growth stages of black gram including floral induction-flowering, pod formation, seed and pod filling and physiological maturity.

### Crop water stress index (CWSI)

To investigate and describe the CWSI, a relationship based on two parameters of (T_c_ − T_a_) and AVPD is presented as follows^10^, and the line obtained by this equation is called the lower baseline (L.L)^6^:

2 $$\left( {T_{c} - T_{a} } \right)_{L.L} = a - b(AVPD) = a - b\left\{ {10 \times {\text{e}}^{{\left[ {\frac{{16.78T_{a} - 116.9}}{{T_{a} + 237.3}}} \right]}} \times \left( {1 - \frac{RH}{{100}}} \right)} \right\}$$

where T_c_ is the canopy cover temperature (°C), T_a_ air temperature (°C), AVPD air vapor pressure deficit (mbar), RH relative humidity (%), a and b are the different constant coefficients for crops and fruit trees. The lower baseline is a special characteristic of each plant and represents the conditions where the plant has no limitations on root water supply, and the air vaporization rate is at its maximum^10^. The upper baseline (U.L) also represents the maximum (T_c_ − T_a_) expected. The upper baseline status is obtained using the following relation^10^:

3 $$\left( {T_{c} - T_{a} } \right)_{U.L} = a + b(AVPG) = a + b\left\{ {e_{s} (T_{a} + a) - e_{s} (T_{a} )} \right\}$$

4 $$e_{s} (T_{a} ) = \left( {0.6108 \times {\text{e}}^{{\left( {\frac{{17.27 \times T_{a} }}{{237.3 + T_{a} }}} \right)}} } \right) \times \left( {\frac{1000}{{101}}} \right)$$

where AVPG is the air vapor pressure gradient (mbar) and coefficients a and b are obtained from the lower baseline (Eq. (2)). The empirical CWSI is also calculated by the following equation^10^:

5 $$CWSI = \frac{{\left( {T_{c} - T_{a} } \right)_{m} - \left( {T_{c} - T_{a} } \right)_{L.L} }}{{\left( {T_{c} - T_{a} } \right)_{U.L} - \left( {T_{c} - T_{a} } \right)_{L.L} }}$$

where (T_c_ − T_a_)_m_ is the difference between the canopy temperature and air temperature (pre-irrigation) at the time of measurement (°C), (T_c_ − T_a_)_L.L_ is the difference between the canopy temperature and air temperature (post-irrigation), which is obtained from the lower baseline equation. (T_c_ − T_a_)_U.L_ is a constant for the upper baseline (post-irrigation).

### Relative water content (RWC)

Relative water content (RWC) of black gram leaves was measured four times during crop growth period for each treatment (with three replications). RWC measurements were performed during four days (before and after irrigation) every other week. On the days of measurement, two to three adult and young leaves in the direction of the sunlight were cut from each plot after T_c_ measurement at the maximum stress time. They were placed in plastic bags and transferred to the laboratory immediately^21^. The RWC values were obtained by the following Eq. :^29^

6 $${\text{RWC}} = \frac{{{\text{F}}_{{\text{W}}} - {\text{D}}_{{\text{W}}} }}{{{\text{T}}_{{\text{W}}} - {\text{D}}_{{\text{W}}} }}$$

where F_W_ is fresh leaf weight (g), T_W_ leaf weight at full turgidity (g) and D_W_ leaf weight when dried in oven (g). The F_W_ value was obtained after the leaves were removed from the crop. The leaves were then immersed in distilled water for four hours. After achieving equilibrium, the leaves were removed by forceps and dried gently and their weight (T_W_) was measured. Finally, the leaves were placed in a paper bag to dry in the oven at 70 °C for 24 h, so that their dry weight (D_W_) is obtained^29,30^.

### Leaf water potential (LWP)

A relatively quick way to measure the water potential in large pieces of crop tissues, such as leaves and stems, is to use a pressure chamber/bomb. This technique assumes that the water pressure within vasculum is close to the average pressure potential of the entire organ because in most cases the osmotic pressure of the vasculum is low. According to Stegman^31^, a pressure bomb measures the compressive or expansive potential of the vasculum. However, because the osmotic potential of the vascular juice is usually insignificant compared to the compressive potential, the negative pressure value in the pressure chamber is often taken as the potential of the entire leaf. The pressure bomb is made of a hollow chamber to accomodiate the leaf specimen. The leaf specimen is used through a gasket to hold the petiole. There is also a pressure capsule that draws compressed air into the chamber. On the other hand, the same chamber is also connected to a barometer. The leaf is placed into the chamber in a way that the petiole remains outside, and as the valve of the compressed air capsule opens, the pressure inside the chamber gradually increases. The leaf wrinkles (wilts) and reaches a pressure point where a drop comes out of the petiole. Just when the first drop comes out of the petiole, the pressure is read on the machine.

The procedure was that in the afternoon, when the LWP reached its lowest (2–3 p.m. local time), the leaf samples, which were completely exposed to sunlight, were selected from each treatment plot (three replications). After each leaf sample was selected, it was cut by a sharp blade cutter and immediately put into the apparatus and the pressure in the chamber was increased by opening the gas valve. As the pressure in the chamber was increased by a handheld magnifying glass, the cut end of the leaf lamina remaining outside the device was carefully observed. This procedure was repeated for other samples and the mean LWP was obtained for three plots in each treatment.

### Soil measurements

#### Soil water (SW)

The SW content is measured either directly (by weighting) or indirectly (profile probe device or PR2). In direct methodology, the mass or volume of water is specifically measured, but in indirect methodology, another factor that is related to the water content is measured first. Then the SW content is estimated based thereupon. The PR2 device is manufactured in two models PR2/4 (with four sensors) and PR2/6 (with six sensors) to measure the SW content in the vertical section. In PR2/6, six sensors are installed on the 1 m bar that enters the soil, and it simultaneously measures the SW content at the depths of 10, 20, 30, 40, 60 and 100 cm. The sensor diameter of the machine is 25.4 mm and the special plastic pipes to be installed in the soil have a diameter of 28 mm. SW content was measured three to four times per week (pre- and post-irrigation)^32^ to the depth of root development at maximum stress times for all black gram treatments. In the present study, a PR2/6 device calibrated with water weight data was used and tubes were installed in the middle of each plot^21^.

### Water retention curve

To measure the soil water retention curve (SWRC) in the laboratory, four intact specimens were taken with two replicates from the soil at layers with the depths of 10–15 cm, and four from the soil at layers with the depths of 30–35 cm by using sampling cylinders with the capacities of 100 cm^3^ and 50 cm^3^. After saturation of the samples, 0, 20, 30, 100, 330, 1000, 3000, 8000 and 15,000 hPa matric suction were applied to the samples using sand box and pressure plate devices. After equilibrium, their average weighted water content was measured^33,34^. The pressure plate consists of a chamber, such as a pressure-cooker, whosepressure could be increased by a compressor. When the pressure reaches the desired potential point, it is relieved through the drain valve and the lid is removed. Soil samples are removed quickly and their mass water content is measured. The specimens are again inserted into the device and the lid is put on and the pressure is increased to the next potential point. The mass moisture is again measured as above at this point.

The average soil porosity was calculated using the equation $1 - (BD/2.65)$ and is considered as saturated moisture content. The SWRC model was fitted to the measured soil water retention data using RETC software. The equation of van Genuchten^35^ is as follows:

7 $$\theta (h) = \theta_{{\text{r}}} + \left( {\theta_{{\text{s}}} { - }\theta_{{\text{r}}} } \right)\left[ {1 + \left( {\alpha h} \right)^{n} } \right]^{{\left[ {\frac{1}{n} - 1} \right]}}$$

where *θ(h)* denotes soil volumetric water content (cm^3^ cm^−3^), *h* is the matric suction of the soil (hPa), *θ*_*r*_ is the residual water content (cm^3^ cm^−3^), *θ*_*s*_ represents saturated SW content (cm^3^ cm^−3^), α is the inverse of suction at the turning point (hPa^-1^) and n is the pore size distribution index (–)^36^.

### Penetration resistance (Q) curve

The mechanical strength of the soil is the maximum resistance of soil to mechanical stresses, without deformation and fracture^37^. The *Q* of soil can be measured by Automatic Micro Penetrometer, which uses a proprietary software called KMP2 to allow the user to obtain accurate data on the *Q* of the soil sample by adjusting the depth and velocity of the cone penetrating into the soil. The selectable diameter of the cone is 1–5 mm. Six velocities varying from 2 to 30 mm min^−1^ were created for the penetration of the cone Penetrometer into the soil sample. The display of *Q* values measured when the cone is inserted into the soil allows the user to capture the data from the logger without storing them on the computer. An automatic recording of *Q* values in text format or ASCI allows the user to read and use the input and output data (results) stored in the Excel software. In the results section, every 0.5 mm penetration in the soil, one piece of data is recorded and reported by the KMP2 software. This section also reports the total average *Q*, the highest and the lowest measured values, and the measurement time.

To measure the soil *Q* characteristic curve (SPRC), five undisturbed soil samples from the 10–15 cm depth layer and five undisturbed soil samples from the 30–35 cm depth received different water levels. To homogenize the water distribution, the 10 soil samples were placed in plastic bags for four weeks. After water equilibrium was obtained, the *Q* values were measured using an in vitro Automatic Micro Penetrometer with a penetration rate of 5 mm min^−1^ in 3 replications with the vertices arranged in a triangle on 10 samples^38^. The water content of soil samples was measured and converted to volumetric moisture content using BD values^21^. The SPRC model was fitted to the measured soil *Q* data using the Solver program. In order to measure SPRC, van Genuchten's^35^ adjusted model was applied in Eq. (8):

8 $$Q = Q_{l} + \left( {Q_{h} - Q_{l} } \right)\left[ {1 + \left( {\alpha_{Q\theta } \theta } \right)^{{n_{Q\theta } }} } \right]^{{\left[ {\frac{1}{{n_{Q\theta } }} - 1} \right]}}$$

In this equation, *Q* is the soil penetration resistance (MPa), *θ *the soil volumetric moisture (cm^3^ cm^−3^), *Q*_*l*_ the lowest soil predicted resistance (MPa), *Q*_*h*_ the highest soil predicted resistance (MPa), *α*_*Qθ*_ (cm^3^ cm^−3^), and *n*_*Qθ*_(–) the fitting parameters of the model related to the turning point and gradient of the function of mechanical resistance to the SW content.

### Model evaluation

To investigate the efficiency of regression models obtained in the results section of the present study to estimate RWC, (T_c_ − T_a_) and LWP, the correlation coefficient (*R*), root mean square error (RMSE), mean absolute error (MAE) and mean relative error (MRE) were used^39^.

9 $$R = \frac{{\sum\limits_{i = 1}^{N} {(O_{i} - \overline{{O_{i} }} )(P_{i} - \overline{{P_{i} }} )} }}{{\sqrt {\sum\limits_{i = 1}^{N} {(O_{i} - \overline{{O_{i} }} )^{2} \sum\limits_{i = 1}^{N} {(P_{i} - \overline{{P_{i} }} )^{2} } } } }}$$

10 $$RMSE = \sqrt {\frac{1}{N}\sum\limits_{i = 1}^{N} {(O_{i} - P_{i} )^{2} } }$$

11 $$MAE = \frac{1}{N}\sum\limits_{i = 1}^{N} {\left| {O_{i} - P_{i} } \right|}$$

12 $$MRE = \frac{1}{N}\sum\limits_{i = 1}^{N} {\frac{{\left| {O_{i} - P_{i} } \right|}}{{O_{i} }}}$$

In these equations, *N* is the number of measurements (samples), *i* index of each model, O_i_ and P_i_ measured and predicted values, and $\overline{O}_{i}$ and $\overline{{P_{i} }}$ mean measured and predicted values. However, the results from the above four criteria sometimes vary in different models^40^. The Ideal Point Error (IPE) index was used to select the best model with the highest accuracy. This index combines the effects of four error criteria and helps choosing the right model. The IPE index is based on identifying the ideal point in the n-dimensional space (n is the number of statistical criteria to evaluate the models), which every models seeks to approach. The ideal point coordinates should be RMSE = 0.0; MAE = 0.0; MRE = 0.0; *R* = 1.0. The IPE index (Eq. (13)) indicates how far the model is from the ideal point^39^.

13 $$IPE_{A} = \left[ {0.25\left( {\left(\frac{{RMSE_{i} - 0.0}}{\max (RMSE)}\right)^{2} + \left(\frac{{MAE_{i} - 0.0}}{\max (MAE)}\right)^{2} + \left(\frac{{MRE_{i} - 0.0}}{{\max \left| {MRE} \right|}}\right)^{2} + \left(\frac{{R_{i} - 1.0}}{1/\max (R)}\right)^{2} } \right)} \right]^{\frac{1}{2}}$$

where, for the model i, max(x) is the maximum value of the x statistic between a groups of study models and is used as a model performance standardization factor for each individual evaluation index. The value of the IPE index varies from zero (best model) to one (worst model), and the closer to zero, the more appropriate the model will be^39^.

A careful examination of the original IPE_A_ equation shows that there is a contradiction in the standardization method applied to each component. In Eq. (13), the first three indices are standardized according to the worst model performance, while the last index (*R*) is standardized according to the best model performance. It should also be noted that standardization of *R* in the original IPEA equation is not designed for negative values^41^. Equation (14) or IPE_B_ represents an improved variant of the original equation, which includes a more generalized and robust standardization procedure for *R* that can accommodate the full range [− 1, + 1]. IPE_B_ is also consistent with the method of standardization with regard to the worst-case model performance. So, the new index eliminates the inconsistency (contradiction) of standardization of the original IPE_A_ equation. This correction can lead to a significant difference between the output of the original IPE_A_ and the IPE_B_, especially for the states containing medium or low *R* values^41^.

14 $$IPE_{B} = \left[ {0.25\left( {\left(\frac{{RMSE_{i} }}{\max (RMSE)}\right)^{2} + \left(\frac{{MAE_{i} }}{\max (MAE)}\right)^{2} + \left(\frac{{MRE_{i} }}{{\max \left| {MRE} \right|}}\right)^{2} + \left(\frac{{R_{i} - 1}}{\min (R) - 1}\right)^{2} } \right)} \right]^{\frac{1}{2}}$$

## Results and discussion

### Baseline equations and CWSI

**Figure 2:**
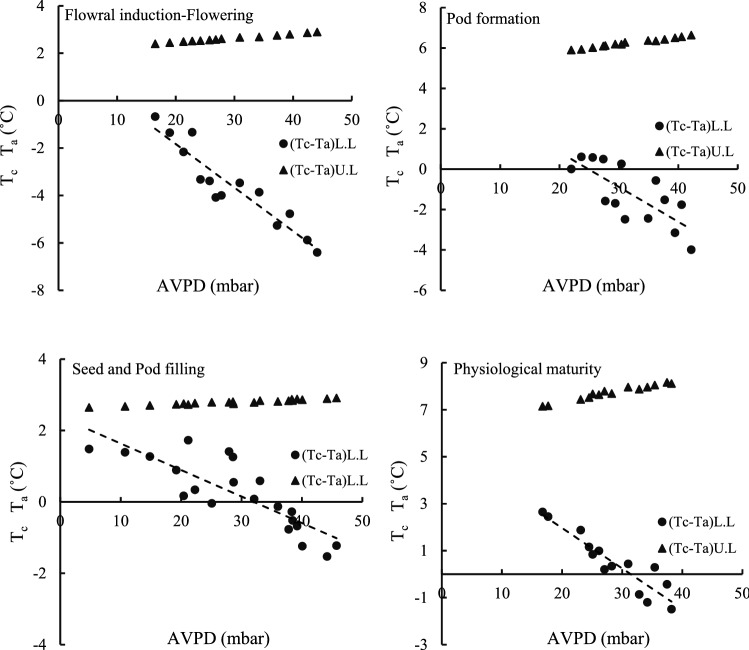
Lower and upper baselines for four stages of black gram growth.

To calculate the lower baseline of the experimental method of Idso et al.^10^, T_c_ of black gram was measured from 8:50 am to 02:50 pm. Lower baseline equations using this experimental method for the four growth stages of black gram (floral induction-flowering, pod formation, seed and pod filling and physiological maturity) are presented for different days after irrigation in Table 6. The correlation between (T_c_ − T_a_) and AVPD is also shown in Fig. 2. According to this figure, the range of AVPD and (T_c_ − T_a_) for the four-stage growth of black gram are, respectively, 4 to 46 mbar and 3 to − 7 °C. The lower baseline equations fitted to the four growth stages can be used in different locations for black gram as long as the AVPD range has a wide range^42^. As AVPD increases, (T_c_ − T_a_) increases (in absolute value) while the rate of increase (T_c_ − T_a_) decreases with time^6^. Examination of the lower baseline relationships showed that the coefficients a and b were different for each growth stage of the black gram. There was also a negative gradient for all four growth stages of crop (Table 6). Due to the differences in the values of these coefficients, one can point to the difference of water uptake potential and also the rate of transpiration during plant growth stages^21^.

**Table 6:** Equations for lower and upper baselines by Idso et al.32 for four stages of black gram growth.

Date	Growth stages	Lower baseline	Upper baseline	R^2^	p-value
July 11 to July 21	Flowral induction-Flowering	(T_a_ − T_c_)_L.L_ = 1.8391 − 0.1836(AVPD)	(T_a_ − T_c_ )_U.L_ = 2.63	0.88	< 0.001
July 25 to August 4	Pod formation	(T_a_ − T_c_ )_L.L_ = 4.2821 − 0.1723(AVPD)	(T_a_ − T_c_ )_U.L_ = 6.25	0.57	< 0.010
August 8 to August 22	Seed and Pod filling	(T_a_ − T_c_ )_L.L_ = 2.3767 −0.0742 (AVPD)	(T_a_ − T_c_ )_U.L_ = 2.79	0.70	< 0.001
August 26 to September 5	Physiological maturity	(T_a_ − T_c_ )_L.L_ = 5.4043 − 0.172 (AVPD)	(T_a_ − T_c_ )_U.L_ = 7.73	0.85	< 0.001

dT_L.L_: lower baseline; dT_U.L_: upper baseline; AVPD: air vapor pressure deficit; *R*^*2*^: coefficient of determination.

The upper baseline values were calculated using the Idso et al.^10^ method for the four growth stages of black gram as 2.63, 6.25, 2.79 and 7.73 °C, respectively (Table 6). According to CWSI-based irrigation scheduling research, it is clear that the upper baseline depends on the crop species, crop variety and climatic conditions of each area^6,21^. The specificity of the lower and upper baselines for each crop indicates that at maximum transpiration, each crop reacts to a variety of stresses (water, salinity, fertility, etc.) and meteorological parameters (temperature, wind speed, relative humidity, etc.); and the transpiration value differs in various crops^21^.

The lower and upper baseline equations at the different dates of the black gram growth period are presented in Table 7. According to this table, the values of a and b coefficients were different at different days of the crop growth for the lower baseline equations, the reasons for which were discussed in the previous section. The *R*^*2*^ of the lower baseline equations ranges from 0.82 to 0.98, and the high value of *R*^*2*^ and the low *P-value* represent acceptable accuracy of the regression equations. It should be noted that the range of the upper baseline equations was from 2.46 to 9.90 °C, which indicates a complete stress condition during the crop growth period.

**Table 7:** Equations for lower and upper baselines at different dates of the black gram growth period.

Date	Lower baseline	R2	Upper baseline
July 13	(T_a_ − T_c_)_L.L_ = 4.4634 − 0.3115 (AVPD)	0.98	(T_a_ − T_c_)_U.L_ = 7.44
July 20	(T_a_ − T_c_)_L.L_ = − 3.811 − 0.2297 (AVPD)	0.97	( T_a_ − T_c_)_U.L_= 6.26
July 27	( T_a_ − T_c_)_L.L_ = 3.5435 − 0.1771 (AVPD)	0.94	( T_a_ − T_c_)_U.L_= 5.22
August 3	( T_a_ − T_c_)_L.L_ = − 4.4407 − 0.1489 (AVPD)	0.91	( T_a_ − T_c_)_U.L_ = 6.19
August 10	(T_a_ − T_c_)_L.L_ = − 4.6014 − 0.1391 (AVPD)	0.93	( T_a_ − T_c_)_U.L_ = 6.31
August 17	(T_a_ − T_c_)_L.L_ = 2.1254 − 0.080(AVPD)	0.82	( T_a_ − T_c_)_U.L_ = 2.46
August 21	( T_a_ − T_c_) _L.L_= − 4.9039 − 0.1347(AVPD)	0.96	(T_a_ − T_c_)_U.L_ = 6.72
August 28	( T_a_ − T_c_)_L.L_ = − 6.4275 − 0.2149(AVPD)	0.97	(T_a_ − T_c_)_U.L_ = 9.90
September 4	( T_a_ − T_c_)_L.L_ = − 4.3143− 0.1268(AVPD)	0.83	(T_a_ − T_c_)_U.L_ = 5.67

(T_c_—T_a_)_L.L_: lower baseline; (T_c_—T_a_)_U.L_: upper baseline; AVPD: air vapor pressure deficit; *R*^*2*^: coefficient of determination.

After formulating the lower and upper baseline equations by Idso’s et al.^10^ method for the four growth stages of black gram (Table 6) and also calculating the mean (T_c_ − T_a_) on the pre-irrigation days (11:50 am to 02:50 pm), CWSI values were calculated for treatments I_1_, I_2_ and I_3_ (Table 8). According to Table 8, the CWSI threshold values for the control treatment (I_3_) in the four growth stages were 0.14, 0.08, 0.22, and 0.15, respectively.Moreover, the mean CWSI values during the growth period of the black gram for the three treatments of 50, 75 and 100% of crop water requirement were calculated to be 0.37, 0.23 and 0.15, respectively. The maximum CWSI for all three treatments occurred on August 8 to August 22 (pod and seed filling stage) and the highest CWSI was related to severe irrigation deficit (I_1_) treatment.

**Table 8:** CWSI threshold and average values during black gram growth period.

Growth stages	I1 (50%)	I2 (75%)	I3 (100%)
Flowral induction-flowering	–	–	0.14
Pod formation	0.28	0.13	0.08
Seed and Pod filling	0.47	0.28	0.22
Physiological maturity	0.35	0.27	0.15
Average	0.37	0.23	0.15

The CWSI empirical method^10^ has been used in various research for plant irrigation management. In the study of CWSI threshold for the soybean, the value of 0.18 was obtained^43^. Also, in other investigations on chili pepper and eggplant under surface drip irrigation, CWSI threshold values were 0.20 and 0.26, respectively^28,44^. It should be noted that so far no research has been done to evaluate water stress index of black gram to be compared with the results of the present study. The higher the SW content, the lower the ambient temperature will be. Thus, the study treatments change the environmental conditions. Meantime, the crops that grow under stress will have different physiological and morphological characteristics. Finally, by increasing water stress, the stomata of the plant are closed and T_c_, and thus CWSI would increase^21,45^.

### Irrigation scheduling using of CWSI

In this study, CWSI was used for irrigation scheduling of black gram (four growth stages). Since the lowest CWSI (0.15) for black gram was observed in the unstressed treatment, this treatment was taken as the basis of irrigation scheduling if the black gram based on the experimental method. The CWSI values obtained using Eq. (5) for the four growth stages of crop are presented in Table 8. Therefore, using the CWSI values and Eq. (15), the equations required for irrigation scheduling of black gram are presented in Table 9 for the four growth stages of the crop in Urmia.

15 $$CWSI_{i} = \frac{{(T_{c} - T_{a} )_{c} - dT_{L.L} }}{{dT_{U.L} - dT_{L.L} }}$$

**Table 9:** Relationships used for black gram irrigation scheduling.

Date	Growth stages	Scheduling relationships
July 11 to July 21	Flowral induction-flowering	(T_a_ − T_c_)_c_ = 1.9498 − 0.1579 (AVPD)
July 25 to August 4	Pod formation	(T_a_ − T_c_)_c_ = 4.4395 c − 0.1585(AVPD)
August 8 to August 22	Seed and Pod filling	(T_a_ − T_c_)_c_ = 2.4676 c − 0.0578 (AVPD)
August 26 to September 5	Physiological maturity	(T_a_ − T_c_)_c_ = 5.7532 c − 0.1462(AVPD)

(T_c_—T_a_)_c_: the permitted difference between the canopy temperature and air temperature; AVPD: air vapor pressure deficit.

The parameters of Eq. (15) have already been described.

To determine the irrigation schedule, the values of T_a_ and RH must first be measured at 11:50 am to 02:50 pm and then the AVPD is calculated. Finally, substituting AVPD in the existing equations Table 9, the allowed (T_c_ − T_a_)_c_ value could be calculated^21^. To determine the irrigation time, we can compare the (T_c_ − T_a_)_m_ (mean values calculated in the field) and (T_c_ − T_a_)_c_ (permissible values), in which case, three conditions occur: 1. If the mean value of (T_c_ − T_a_)_m_ is less than (T_c_ − T_a_)_c_, it’s too soon to irrigate the crop, 2. If greater than (T_c_ − T_a_)_m_ id larger than (T_c_ − T_a_)_c_, the irrigation has been missed and in the third case, if (T_c_ − T_a_)_c_ and (T_c_ − T_a_)_m_ are equal, it is to time for irrigation^46^.

### Relations between plant and soil indices

#### Linear regression relationships–univariate

**Figure 3:**
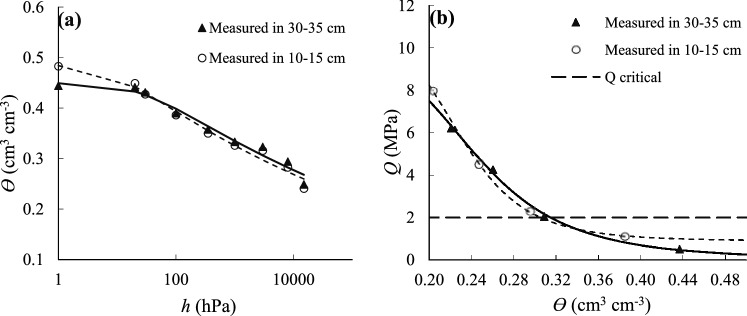
van Genuchten^35^ function fitted to the SWRC (**a**) and modified van Genuchten^35^ model fitted to the SPRC (**b**) data; well-known critical *Q* = 2.0 MPa is shown on the graph.

The use of classical methods to estimate and monitor the water depletion in the crop-soil system requires measurement of SW content, crop properties or climatic variables. Unless a large number of samples are produced and interpreted, these methods are time-consuming and provide a poor description of the overall situation of the field due to producing point data^47^. Van Genuchten's^35^ model fitting parameters in layers of 10 − 15 cm (surface) and 30 − 35 cm (lower) depth are presented in Table 10 for the soil water retention curve (SWRC) of the black gram. According to Table 10, van Genuchten's^35^ model fitted well to the in vitro data because high values of *R*^*2*^ were obtained in the surface and lower layers (Fig. 3a).

**Table 10:** Parameters of van Genuchten^35^ model for the SWRC (a) and modified van Genuchten^35^ model for the SPRC (b) fitted to the measured data.

(a)					
Soil layer (cm)	*θ*_r_	*θ*_s_	*α*	*n*	*R*^*2*^
	cm^3^ cm^−3^		hPa^-1^	(–)	
10–15	0.000	0.488	0.123	1.084	0.982
30–35	0.000	0.450	0.035	1.083	0.972

(b)					
Soil layer (cm)	*Q*_*l*_	*Q*_*h*_	*α*_*Qθ*_	*n*	*R*^*2*^
	MPa		cm^−3^ cm^3^	(–)	
10–15	0.897	10.810	4.465	7.826	0.999
30–35	0.047	9.907	4.284	5.972	0.998

*θ*_*r*_ and *θ*_*s*_: residual moisture and soil saturation, respectively; *α*: inverse suction in the turning point (air entry point); *n*: pore size distribution index; *Q*_*l*_ and *Q*_*h*_: lowest (wet) and maximum (dry) predicted soil penetration resistance, respectively; *α*_*Qθ*_ and *n*_*Qθ*_: fitness parameters of the model related to the turning point and slope of the mechanical strength function against soil water; *R*^*2*^ is coefficient of determination.

**Table 11:** Linear different models and statistical parameters for RWC estimating.

Model	Variables	R	RMSE	MAE	MRE	N	p-value	IPEA	IPEB
		(%)	(–)	(–)	(–)			(–)	(–)
1	(T_c_ − T_a_)	44.1	0.027	0.022	0.028	64	0.000	0.806	0.865
2	SW	25	0.029	0.023	0.029	64	0.045	0.866	0.964
3	Q	23.1	0.029	0.023	0.029	64	0.064	0.873	0.975
4	RH	18.7	0.029	0.023	0.03	64	0.141	0.888	1.000
5	(T_c_ − T_a_), SW	48.1	0.026	0.021	0.027	64	0.000	0.795	0.847
6	(T_c_ − T_a_), Q	47.4	0.026	0.021	0.027	64	0.000	0.797	0.85
7	(T_c_ − T_a_), RH	44.6	0.027	0.021	0.027	64	0.001	0.799	0.858
8	SW, Q	25.5	0.029	0.023	0.029	64	0.124	0.865	0.961
9	SW, RH	30.7	0.029	0.022	0.029	64	0.048	0.846	0.932
10	Q, RH	29.8	0.029	0.022	0.028	64	0.058	0.845	0.934
11	(T_c_ − T_a_), SW, Q	48.4	0.026	0.021	0.027	64	0.001	0.795	0.846
12	(T_c_ − T_a_), SW, RH	48.6	0.026	0.021	0.027	64	0.001	0.788	0.839
13	(T_c_ − T_a_), Q, RH	48	0.026	0.021	0.027	64	0.001	0.789	0.786
14	(T_c_ − T_a_), SW, Q, RH	48.8	0.026	0.021	0.027	64	0.003	0.787	0.783
15	SW, Q, RH	30.8	0.028	0.022	0.029	64	0.108	0.847	0.875

*R* is correlation coefficient; RMSE: root mean square error; MAE: mean absolute error; MRE: mean relative error; IPE: Ideal Point Error; *N* is the number of measurements.

**Table 12:** Linear different models and statistical parameters for (T_c_ − T_a_) estimating.

Model	Variables	R	RMSE	MAE	MRE	N	p-value	IPEA	IPEB
		(%)	(°C)	(°C)	(–)			(–)	(–)
1	SW	50.5	2.47	1.97	− 0.43	140	0.000	0.740	0.771
2	Q	43.8	2.57	2.03	− 0.44	140	0.000	0.769	0.807
3	RH	12.1	2.84	2.30	− 0.29	140	0.154	0.817	0.902
4	AVPD	11.9	2.84	2.31	− 0.40	140	0.16	0.853	0.935
5	SW, Q	50.9	2.46	1.98	− 0.41	140	0.000	0.734	0.765
6	SW, RH	50.6	2.46	1.97	− 0.40	140	0.000	0.730	0.761
7	SW, AVPD	55.4	2.38	1.87	− 0.56	140	0.000	0.785	0.809
8	SW, Q, RH	51.0	2.46	1.98	− 0.40	140	0.000	0.728	0.759
9	SW, Q, AVPD	56.0	2.37	1.87	− 0.53	140	0.000	0.768	0.791
10	Q, RH	44.6	2.56	2.02	− 0.39	140	0.000	0.745	0.783
11	Q, AVPD	47.7	2.51	1.99	− 0.55	140	0.000	0.809	0.840
12	RH, AVPD	47.7	2.51	1.99	− 0.27	140	0.000	0.688	0.724
13	SW, Q, RH, AVPD	72.9	1.96	1.55	− 0.30	140	0.000	0.559	0.571
14	SW, RH, AVPD	72.9	1.96	1.55	− 0.30	140	0.000	0.558	0.571
15	Q, RH, AVPD	69.8	2.05	1.62	− 0.31	140	0.000	0.582	0.597

*R* is correlation coefficient; RMSE: root mean square error; MAE: mean absolute error; MRE: mean relative error; IPE: Ideal Point Error; *N* is the number of measurements.

**Table 13:** Linear different models and statistical parameters for LWP estimating.

Model	Variables	R (%)	RMSE (bar)	MAE (bar)	MRE (–)	N	p-value	IPEA (–)	IPEB (–)
1	(T_c_ − T_a_)	7.9	2.84	2.24	− 0.270	28	0.689	0.884	0.982
2	SW	15.1	2.82	2.22	− 0.271	28	0.442	0.877	0.961
3	Q	9.7	2.84	2.21	− 0.270	28	0.624	0.880	0.975
4	RWC	35.3	2.67	2.11	− 0.247	28	0.065	0.816	0.869
5	RH	1.9	2.85	2.22	− 0.270	28	0.926	0.885	0.995
6	(T_c_ − T_a_), SW	15.9	2.81	2.22	− 0.270	28	0.728	0.875	0.958
7	(T_c_ − T_a_), Q	11.3	2.83	2.22	− 0.270	28	0.850	0.880	0.972
8	(T_c_ − T_a_), RWC	35.8	2.66	2.14	− 0.249	28	0.180	0.821	0.874
9	(T_c_ − T_a_), RH	8.3	2.84	2.24	− 0.271	28	0.918	0.886	0.983
10	SW, Q	29.2	2.72	2.12	− 0.252	28	0.328	0.831	0.893
11	SW, RWC	35.9	2.66	2.11	− 0.249	28	0.179	0.817	0.870
12	SW, RH	16	2.81	2.20	− 0.267	28	0.723	0.870	0.953
13	Q, RWC	35.4	2.66	2.11	− 0.248	28	0.188	0.817	0.871
14	Q, RH	10.2	2.83	2.20	− 0.268	28	0.878	0.876	0.970
15	RWC, RH	36.2	2.66	2.11	− 0.250	28	0.174	0.818	0.870
16	(T_c_ − T_a_), SW, Q	29.8	2.72	2.11	− 0.251	28	0.517	0.828	0.890
17	(T_c_ − T_a_), SW, RWC	36.2	2.66	2.14	− 0.251	28	0.330	0.822	0.874
18	(T_c_ − T_a_), SW, RH	16.5	2.81	2.21	− 0.268	28	0.880	0.872	0.955
19	(T_c_ − T_a_), SW, Q, RWC, RH	41.8	2.59	2.09	− 0.243	28	0.478	0.800	0.844
20	(T_c_ − T_a_), SW, Q, RWC	40.5	2.60	2.12	− 0.243	28	0.368	0.805	0.851
21	(T_c_ − T_a_), SW, Q, RH	29.9	2.72	2.12	− 0.252	28	0.691	0.829	0.890
22	SW, Q, RWC, RH	41.1	2.6	2.06	− 0.241	28	0.349	0.795	0.841
23	SW, Q, RWC	40.1	2.61	2.09	− 0.242	28	0.232	0.801	0.848
24	SW, Q, RH	29.2	2.72	2.12	− 0.253	28	0.535	0.831	0.894
25	Q, RWC, RH	36.2	2.66	2.10	− 0.249	28	0.329	0.816	0.868

*R* is correlation coefficient; RMSE: root mean square error; MAE: mean absolute error; MRE: mean relative error; IPE: Ideal Point Error; *N* is the number of measurements.

In this study, the I_4_ treatment was considered for soil ventilation porosity^21^ and sometimes, just because the measurements (canopy temperature) have been made post-irrigation does not mean there are unstressed conditions and in this case, the data of the treatment that received the highest amount of water can be used for the rest of the treatments. The critical level of ventilation porosity is about 10% the ventilation porosity of root growth^48^, and if the soil *Q* values are between 1.5 and 4 MPa, it restricts root development and 2 MPa (critical *Q*) is the most acceptable value^48^. The soil *Q* content increased drastically by decreasing the soil moisture, thereby causing the plant to be simultaneously affected by two types of stress, i.e. soil water deficit and soil *Q* increase^49^.

The fitting parameters of van Genuchten's^35^ model in the surface and lower layers (Fig. 3b) for the soil *Q* curve (SPRC) are also presented in Table 10 and high values for *R*^*2*^ are obtained due to a better control of conditions and uniform water distribution in the samples taken from the two layers^38^. In Fig. 3b, the well-known critical *Q* has been identified, and the critical *Q* value of 2 MPa was observed for the black gram in the lower layer at about 0.315 cm^3^ cm^−3^ and at the surface layer at about 0.305 cm^3^ cm^−3^; so, the water contents of these two layers were close together. In the SWRC of the black gram (Fig. 3a) there was no significant difference in the pore size distribution, so the SWRC (Fig. 3a) and SPRC (Fig. 3b) curves were overlapped and are similar to one another.

**Figure 4:**
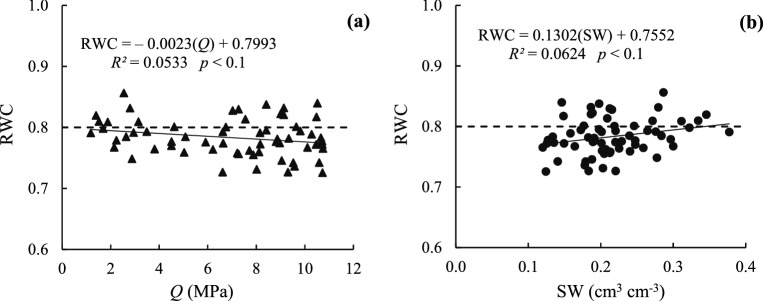
Linear relations betw**e**en RWC and *Q* (**a**) and RWC and SW (**b**) indices.

**Figure 5:**
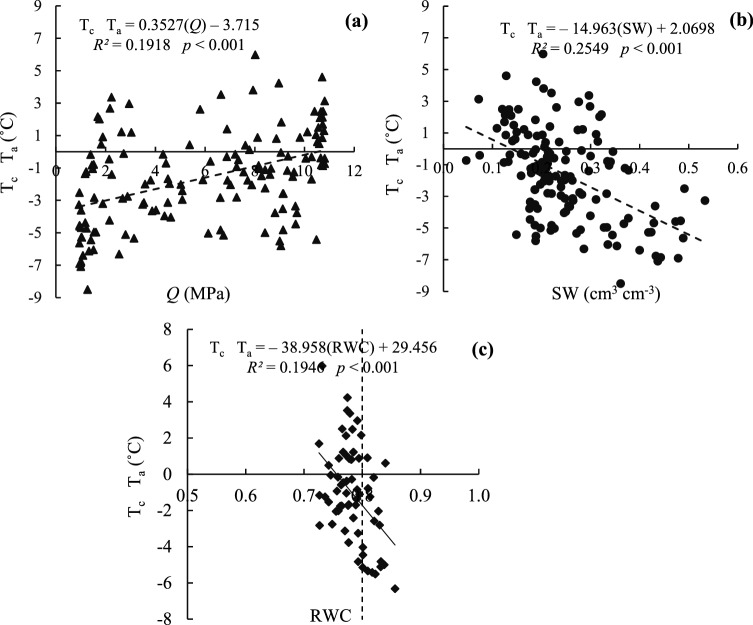
Linear relations between (T_c_ − T_a_) and *Q* (**a**), (T_c_ − T_a_) and SW (**b**), and (T_c_ − T_a_) and RWC (**c**) indices.

Water and temperature stresses are among the most important abiotic stresses that occur at different growth stages in arid and semiarid areas^50^. Unfortunately, in recent years crop production (such as rice, maize and wheat) has declined sharply in many parts of Asia due to rising water stress, so breeders are more likely to cope with this problem through selecting drought resistant cultivars with high water use efficiency. They use agronomic, physiological and biological methods for this purpose^51^. Regression equations were extracted for plant and soil indices measured simultaneously at maximum stress hours during the growth period of black gram (one of drought tolerant crops). The relationship between RWC and soil indices (*Q* and SW), relationship between (T_c_ − T_a_) and soil and plant indices (*Q*, SW and RWC), the relationship between leaf water potential (LWP) and soil and plant indices (*Q*, SW and RWC) as well as with the plant indices and meteorological parameters (T_c_ − T_a_) are observed (Figs. 4, 5 and 7).

According to the Hofler diagram^30^ and the critical limit of RWC (0.80), if the value of 0.80 is assumed as the critical limit of RWC in the black gram, according to Fig. 4a,b, the critical value of *Q* and SW is − 0.3 MPa and 0.34 cm^3^ cm^−3^ (matric potential = 520 hPa). The critical values of *Q* (with negative coefficient) and SW obtained for black gram are not reasonable values since *Q* cannot be less than zero. Therefore, it is not acceptable. Moreover, the accuracy of regression equations obtained for black gram is low. It should be noted that the Hofler diagram has been obtained for specific crops such as maize and wheat, while the black gram is a drought tolerant crop. According to Fig. 4a,b, an increase in the soil *Q* (with decreasing SW) decreased the RWC value of the leaf only slightly (with low gradient). The permissible (T_c_ − T_a_)_c_ value was also calculated for the entire growth period of black gram and was about − 0.036 °C, which can be taken as the critical value (T_c_ − T_a_). Consequently, the critical values of *Q*, SW and RWC for the entire growth period are 10.43 MPa, 0.14 cm^3^ cm^−3^ and 0.76, respectively. It should be noted that the values of SW and *Q* were lower and higher than expected, respectively, although black gram is a drought tolerant plant. So, it is advisable to repeat these values for other conditions. According to Fig. 5a,b, changes in (T_c_ − T_a_) relative to *Q* and SW are almost linear, that is, as the SW decreases (increasing *Q*), the value of (T_c_ − T_a_) increases. According to Fig. 5c, by decreasing RWC, (T_c_ − T_a_) increases and therefore, it would increase by decreasing AVPD.

According to Khorsand et al.^21^ study on maize, with loss of soil water, the RWC of the crop lowers (higher gradient), but some crops such as black gram that are more tolerant lower their RWC less by reducing their transpiration or managing their water more effrectively. In other words, their RWC decreases with a lower gradient (Fig. 4b), indicating that the leaf water content is lost less rapidly. RWC is one of the important physiological indices that has good correlation with resistance to drought stress. By increasing the drought stress, the RWC of the leaf decreases^52^. Cultivars that are able to maintain a greater leaf RWC under reduced SW content will have greater resistance to water loss. In other words, they have a higher ability to absorb and retain water. In a study by Siddique et al.^53^, increased drought stress reduced the RWC value of wheat, and typically, drought tolerant cultivars show higher RWC than cultivars susceptible to drought stress.

According to the field data obtained from black gram cultivation in West Azerbaijan Province, this crop is highly resilient tolerance to water deficit stress and the ratio of root to the shoot in higher in this crop. Typically, species with higher R/S ratios are more susceptible to drought stress. Singh et al.^54^ stated that crops with longer roots, a higher number of lateral roots, root length and higher R/S are more resistant to water deficit and drought stress. The root of black gram (leguminosae), which is a dicot, is right-sided, with two important characteristics: (1) Absorbing water from higher depth, and (2) water-nutrition retaining root. A decrease in leaf RWC can be due to a decrease in the amount of water absorbed from the soil by the roots or due to evaporation from the stomata^55^. Moreover, the high RWC in water deficit conditions can be related to the behavior of the stomata and root system of the crop^56^, because retaining the water content of the crop requires deep roots to absorb water^57^. Drought-tolerant plants use several features to reduce transpiration:

1. Morphological features including: (a) Number of stomata; (b) Size of stomata; (c) Location of stomata; (d) The fluffs existing on the leaf surface; (e) Leaf turndown (epinasty or downward bending in leaves)^58^: We observed this phenomenon (epinasty) in the black gram at the peak of the maximum stress hour (03:00 pm), and this may be another reason for the decrease in transpiration and non-variation of RWC; (f) Leaf angle; (g) Wax layer on leaf surface.
2. Physiological features including: (a) Closure of stomata: With higher production of Abscisic Acid (ABA), the wild species can close their stomata more quickly and drastically (transpiration decreases, i.e. the RWC does not change significantly). A sharp decrease in the stomatal conductance with slight variations of RWC indicates that the signals from the root in drought stress condition are the probable cause of stomatal closure and decrease in photosynthesis, and this chemical signal is ABA^59^. When water deficiency condition is over, ABA disappears, resulting in potassium (K^+^) ions entering the guar cells and increasing the concentration of substances within the cell, and thus, a reverse phenomenon^60^. (b) An increase in the carbohydrates soluble in plant tissues (decrease in transpiration); (c) increase in proline amino acid concentration (decrease of transpiration); (d) increase in the phenolic compounds in plants that prevents any increase of plant temperature, thereby decreasing transpiration.

**Figure 6:**
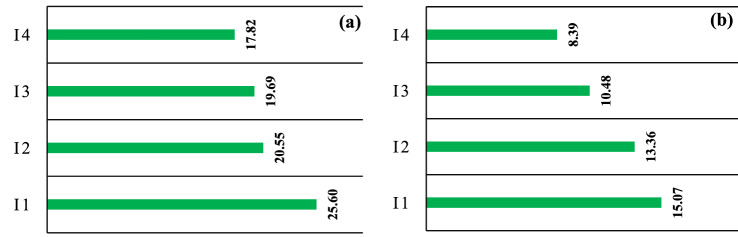
Comparison of mean total phenolics (**a**) and mean flavonoid content (**b**) in black gram seeds in different irrigation treatments.

Higher RWC in leaves may be obtained through osmotic regulation or the ability of the root to absorb water^61^. There are many antioxidant compounds in plants, including phenolic, flavonoids, carotenoids and benzoic acid^62^. Phenolic compounds are one of the most widespread phytochemical groups that are of great morphological and physiological importance in the plants. These compounds are calssified based on their structural diversity in nature, the most important of which are flavonoids, phenolic acids and tannins^63^. In the present study, the total phenol and flavonoid content in different irrigation regimes (three iterations) of black gram were a determined. According to the results, the highest total phenol content (22.6 mg of gallic acid per 100 g of seed dry matter) was observed in 50% water requirement (I_1_) treatment and the lowest content (17.82 mg of gallic acid in 100 g of seed dry matter) in 125% water requirement (I_4_) treatment (Fig. 6a). The high content of phenolic compounds is the main reason for the high antioxidant activity of some extracts, including polar extracts^64^. Phenolic compounds have reductive characteristics that allow them to act as reductants and hydrogen donors and reducers of singlet oxygen^65^. The antioxidant activity of plants depends on different characteristics such as genotype, climate, growing season, geographical location, soil type and storage conditions^66^.

According to the results, the highest flavonoid content (15.07 mg of quercetin per 100 g of seed dry matter) was obtained in the 50% water requirement (I_1_) treatment and the lowest content (8.39 mg of quercetin per 100 g of seed dry matter) in 125% water requirement (I_4_) treatment (Fig. 6b). Flavonoids can prevent oxidative stresses; that is, they can scavenge the reactive oxygen species. Evaluation of rapeseed flavonoid content under water stress conditions showed that it increased in the plant as a secondary metabolite^67^.

In general, the results showed that water stress stimulates the phenolic compounds production factors to increase its content in black gram seeds, which could also explain the decrease in the transpiration and non-variation of RWC in this crop. Studying the effect of drought stress on rosemary and lemon balm, Munné-Bosch and Alegre^68^ showed that RWC decreased in these plants by 40% and 34%, respectively. The reason for the decrease in RWC under drought stress is that during the stress period, the transpiration rate is higher than the water absorption of the plant and, as a result of disruption of the water balance of the plant, RWC decreases and this decrease will in turn close the stomata and reduce the stomatal conductance^69^. RWC is one of the important, reliable and widely used indices for identifying cultivars that are tolerant or susceptible to drought stress. A high relative amount of water means the leaf's ability to retain more water under stress. Tolerant species keep their water content at a higher level. Drought tolerant plants and cultivars usually have higher RWCs under drought stress^70^, so plants with high RWCs are suitable for arid areas because they can retain more water without closing their stomata^71^.

**Figure 7:**
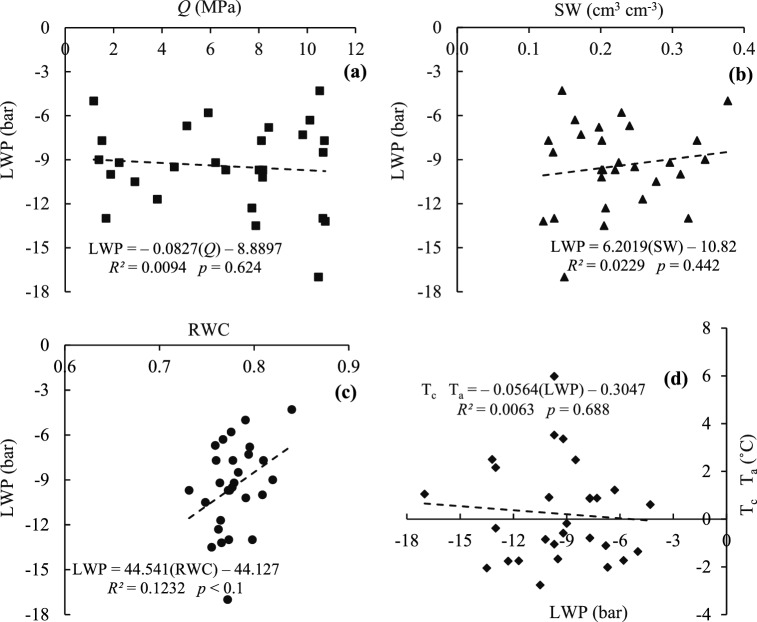
Linear relations between LWP and *Q* (**a**), LWP and SW (**b**), LWP and RWC (**c**), and (T_c_ − T_a_) and LWP (**d**) indices.

RWC is one of several methods to measure water content of the crop that is closely related to LWP and has been reported as an important indicator of drought stress in leaves^72^. LWP was used to measure the leaves of black gram with a pressure bomb, and in Fig. 7, it can be observed that it is correlated with other indices (soil *Q*, SW content, RWC and (T_c_ − T_a_)). The pressure bomb method can measure the water potential of plant tissue in a short time (minutes), but LWP determination needs high accuracy because it is strongly influenced by location, leaf age, radiation and time of sampling or measuring. Therefore, measures should be undertaken to harvest the mature leaves from certain locations at certain times of the day. Although many devices are available in the market for this type of measurement, due to technical problems farmers often do not use this type of device. Meanwhile, due to being time consuming, and the precision and training required, these devices are not widely used.

According to Fig. 7a,b, LWP decreased as soil *Q* increased, and as the water availability of black gram increased (increasing *Q*), LWP decreased, which is consistent with the results of Bajji et al.^73^ It should be noted that the plant used in their research was wheat. The high variability of the measured LWP data is one of the disadvantages of this crop index. For example, for the 75% water requirement (I_2_) treatment, values of − 6, − 9 and − 12.5 bar for LWP were measured on one day (August 21). Also according to Fig. 7c,d, LWP decreased by decreasing RWC of black gram leaves, and by decreasing LWP, the (T_c_ − T_a_) decreased. RWC decreases by increasing moisture stress due to decrease in LWP and decrease in water uptake from roots in drought conditions^53,74^. The decrease in the RWC of leaves can be due to the decrease in SW content and consequently the decrease of LWP due to increased drought stress.

### Linear regression relationships–multivariate

Since univariate-linear regression equations between plant and soil indices were not sufficiently accurate for the black gram, in this section, univariate and multivariate linear regression equations were extracted for the parameters simultaneously measured at a given date for this plants were (peak stress hours) by using the IBM SPSS Statistics 25 software. To estimate RWC (independent parameters including (T_c_ − T_a_), SW, *Q* and RH), (T_c_ − T_a_) (independent parameters including SW, *Q*, RH and AVPD) and LWP (independent parameters including (T_c_ − T_a_), SW, *Q*, RWC and RH) were presented in linear regression models as 15, 15 and 25, respectively. In the present study, the original IPE_A_ index and the new IPE_B_ index were used to estimate the RWC, (T_c_ − T_a_) and LWP and to select the most accurate model. The best models for estimating RWC, T_c_ − T_a_ and LWP are presented in Eqs. (16), (17) and (18), respectively (Tables 11, 12, 13). In the present study, the original IPE_A_ index and the new IPE_B_ index were used to estimate the RWC, (T_c_ − T_a_) and LWP and to select the most accurate model. Their results are presented in Tables (11), (12) and (13). And the best models for estimating RWC (Model 14), T_c_ − T_a_ (Model 14) and LWP (Model 22) are presented in Eqs. (16), (17) and (18), respectively.

16 $$RWC = - 0.004(T_{c} - T_{a} ) + 0.199(SW) + 0.002(Q) + 0.0005(RH) + 0.715$$

17 $$(T_{c} - T_{a} ) = - 16.693(SW) - 0.511(RH) - 0.522(AVPD) + 32.813$$

18 $$LWP = 40.756(SW) + 0.848(Q) + 40.489(RWC) + 0.085(RH) - 56.859$$

## Conclusions

In the present study, plant and soil indices were used to irrigation scheduling and water stress management of black gram, using all effective parameters on evapotranspiration, water uptake by plants and the effect of soil physical properties to select appropriate management practices. The current study was performed using plant and soil indices to remove the limitations of experimental CWSI. Also, utilizing the regression relationships extracted between plant and soil indices during the growth period of black gram, the water status of black gram can be determined by only measured T_c_ and without trying to measure RWC, LWP, SW and *Q* of the soil. It is also worth noting that among these indices, the soil *Q* can be measured rapidly on the farm, because the soil *Q* measuring device is portable and the farmer can easily carry it to the field. Therefore, soil *Q* is a useful indicator for determining irrigation schedule and improving irrigation management^21^. Bulmer and Simpson^75^ also emphasized that, regardless of the gradual or sudden impact of soil *Q*, determining the critical mechanical strength during root growth could be useful in soil water management. According to the field data obtained from black mish planting site, this plant species shows high tolerance to water stress and can be used as a low-water plant due to the climatic conditions of Iran (Urmia region) which is going towards drier each year as an alternative crop. The non-economically of most of the high-produced agricultural products in the Lake Urmia basin, in addition to the problem of farmers' livelihoods, but also has an important role in creating the Urmia lake crisis. Therefore, promoting the cultivation of medicinal plants is a solution on the Urmia Lake crisis. So according to the results, this plant can also be cultivated as one of the low-water medicinal plants in Urmia Lake basin. Another advantage of this study is that the farmer can use the extracted regression relationships between soil and plant indices based on his existing facilities and the critical limits obtained for black mish irrigation.

## Data Availability

The data that support the findings of this study are openly available.

## Abbreviations

AVPD: Air vapor pressure deficit (mbar)
AVPG: Air vapor pressure gradient (mbar)
a and b: Intercept a and slope b are the linear regression parameters of (T_c_ − T_a_)_L.L_ on AVPD (–)
BD: Bulk density (g cm^−^^3^)
CWSI: Crop water stress index (–)
DW: Weight of leaf dried in oven (g)
es(T_a_): Air vapor pressure at T_a_ (mbar)
ET_c_: Crop evapotranspiration (mm)
ET_o_: Reference evapotranspiration (mm)
FC: Field capacity (cm^3^ cm^−^^3^)
FW: Fresh leaf weight (g)
h: Soil matric suction (hPa)
IPE: Ideal point error (–)
IRT: Infrared thermometer (°C)
K_c_: Crop coefficient (–)
LWP: Leaf water potential (bar, MPa)
MAE: Mean absolute error ((–), °C, bar)
MRE: Mean relative error (–)
n: Pore size distribution index (–)
N: Number of measurements (instances) (–)
O_i_ and P_i_: Observed and predicted values (–)
$\overline{O}_{i}$ and $\overline{{P_{i} }}$: Mean of the observed and predicted values (–)
PR2: Profile probe (V%)
PWP: Permanent wilting point (cm^3^ cm^−^^3^)
Q: Penetration resistance (MPa)
Q_h_: Maximum (dry) predicted soil penetration resistance (MPa)
Q_l_: Lowest (wet) predicted soil penetration resistance (MPa)
R2: Coefficient of determination (–)
R: Correlation coefficient (%)
RH: Relative humidity (%)
RMSE: Root mean squared error ((–), °C, bar)
RWC: Relative water content (–)
SPRC: Soil penetration resistance curve (–)
SW: Soil water (cm^3^ cm^−^^3^)
SWRC: Soil water retention curve (–)
T_a_: Air temperature (°C)
T_c_: Canopy temperature (°C)
(T_c_ − T_a_): Difference between the canopy temperature and air temperature (°C)
(T_c_ − T_a_): Permitted difference between the canopy temperature and air temperature (°C)
(T_c_ − T_a_) _L.L_ = dT_L.L_: Lower baseline (°C)
(T_c_ − T_a_)_m_ = dT_m_: Difference between the T_c_ and T_a_ (before irrigation) during measurement (°C)
(T_c_ − T_a_) _U.L_ = dT_U.L_: Upper baseline (°C)
TW: Leaf weight in full turgid (g)
α: Inverse suction in the turning point (air entry point) (hPa^−^^1^)
α_Qθ_ and n_Qθ_: Fitness parameters of the model related to the turning point and slope of the mechanical strength function against soil water (cm^3^ cm^−^^3^ and (–))
θ_r_: Residual water content (cm^3^ cm^−^^3^)
θ_s_: Water content at the saturation (cm^3^ cm^−^^3^)
